# Profoxydim in Focus: A Structural Examination of Herbicide Behavior in Gas and Aqueous Phases

**DOI:** 10.3390/molecules29184371

**Published:** 2024-09-14

**Authors:** María Cobos-Escudero, Paula Pla, Álvaro Cervantes-Diaz, José Luis Alonso-Prados, Pilar Sandín-España, Manuel Alcamí, Al Mokhtar Lamsabhi

**Affiliations:** 1 Unit of Plant Protection Products, Instituto Nacional de Investigación y Tecnología Agraria y Alimentaria (INIA-CSIC), Carretera de La Coruña Km. 7, 28040 Madrid, Spain; maria.cobos@inia.csic.es (M.C.-E.); alvaro.cervantes@inia.csic.es (Á.C.-D.); prados@inia.csic.es (J.L.A.-P.); 2 Departamento de Química, Facultad de Ciencias, M13, Universidad Autónoma de Madrid, 28049 Madrid, Spain; paula.pla@uam.es (P.P.); manuel.alcami@uam.es (M.A.); 3 Institute for Advanced Research in Chemical Sciences (IAdChem), Universidad Autónoma de Madrid, 28049 Madrid, Spain; 4 Instituto Madrileño de Estudios Avanzados en Nanociencias (IMDEA–Nanociencia), 28049 Madrid, Spain

**Keywords:** profoxydim, pesticide, isomerization, tautomerization, DFT

## Abstract

This study investigates the chemical structure of profoxydim, focusing on its E–isomer, the main commercial form. The research aimed to determine the predominant tautomeric forms under various environmental conditions. Using proton and carbon–13 NMR spectroscopy alongside theoretical modeling, we examined tautomers and their conformers in different solvents (MeOD, DMSO, CDCl_3_, benzene) to mimic gas and aqueous phases. The findings reveal that the enolic form dominates in the gas phase, while the ketonic form prevails in aqueous environments, providing key insights into the herbicide’s environmental behavior. We also observed an isomeric transition from E to Z under acidic conditions, which could affect profoxydim’s reactivity in natural environments. The theoretical calculations indicated that in acidic conditions, the E and Z forms are nearly degenerate, with the E form remaining dominant in neutral environments. Additionally, QSAR models assessed the toxicity of various tautomers, revealing significant differences that could impact bioactivity and environmental fate. This research offers crucial insights into the structural dynamics of profoxydim, contributing to cyclohexanedione chemistry and the development of more effective herbicides.

## 1. Introduction

The field of cyclohexane–1,3–dione ether chemistry has been a rich ground for the development of herbicides that exhibit strong and selective action against grass species. These herbicides operate by inhibiting the activity of acetyl–coenzyme A carboxylase, an essential enzyme in the biosynthesis of fatty acids [1]. This inhibition leads to symptoms such as reduced plant growth and eventual plant death. To this end, eight cyclohexanedione (CHD) herbicides have been introduced to the market, with profoxydim (BAS 625H), marketed under the trade name Aura^®^ by BASF in 2000, being the latest. Profoxydim, chemically known as ((1EZ)–N–[(2RS)–2–(4–chlorophenoxy)propoxy]butanimidoyl)–3–hydroxy–5–[(3RS)–thian–3–yl]cyclohex–2–en–1–one) (98.8% purity), has been particularly effective in selectively targeting grass weeds like barnyard grass (Echinochloa crus–galli) without harming rice crops [1,2]. Representing the pinnacle of research in this domain, profoxydim stands out for its combination of high efficacy, exceptional selectivity, and a positive toxicological and ecotoxicological profile [2,3,4,5], making it one of the most relevant new-generation rice herbicides [2,6,7].

The effectiveness of herbicides can be significantly influenced by environmental factors [8]. For cyclohexanedione (CHD) herbicides, pH has been identified as a critical factor affecting their performance [9]. These herbicides are classified as weak acids, possessing pKa values below 5, which means they can easily become ionized [10]. When the pH of the spray solution exceeds the herbicide’s pKa value, the ionized form of the herbicide becomes more prevalent. It is the protonated form of the herbicide; however, it can more efficiently penetrate the plant’s cuticle and adsorb more swiftly compared to its ionized counterpart [11,12,13]. Consequently, the pH of the water used can impact the herbicide’s efficacy, phytotoxicity, and overall effectiveness, making it a vital consideration in planning and executing weed management programs. This aspect is particularly crucial for profoxydim, an herbicide used in rice cultivation, where the crop fields are often submerged in water. Adjusting for pH can therefore enhance profoxydim’s effectiveness in such waterlogged conditions, ensuring optimal weed control and minimal impact on the rice crop.

Current concerns regarding pesticide use prominently include their environmental fate, due to potential risks to human and animal health, and broader ecological impacts. This presents a significant challenge for analytical and environmental chemists. Once deployed in agricultural settings, pesticides can break down, leading to the generation of degradation products (DPs) and metabolites. These by-products may exhibit greater persistence, toxicity, or mobility in the environment compared to their original compounds [14,15,16,17]. The environmental behavior of these DPs can significantly diverge from that of their parent substances, often due to substantial differences in their physicochemical and toxicological properties. The latest research into the degradation of profoxydim in paddy soil and water has revealed the enhanced stability of several novel DPs, which have been identified for the first time in soil [18,19]. While it has been established that CHD herbicides undergo rapid degradation in natural settings, the specific chemical structures and characteristics of their resulting DPs have not been extensively examined or understood.

Within this research framework, the goal is to conduct an in–depth examination of the structural characteristics of profoxydim, with the aim of elucidating its reactivity. This understanding will facilitate explanations of the herbicide’s behavior in environmental contexts and enhance predictions regarding its degradation products and pathways. Investigating profoxydim’s structure in various solvents such as water and octanol will enable predictions about its environmental behavior.

**Figure 1:**
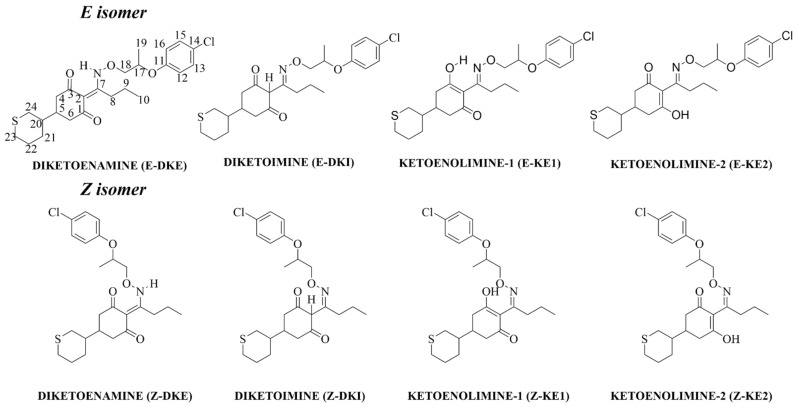
Scheme of the different profoxydim’s tautomers and isomers considered in this study.

The structural basis of CHD herbicides includes an alkoxyaminoalkylidene group attached to the second position of the cyclohexanedione ring, a feature crucial for their herbicidal effectiveness. Meanwhile, modifications at the fifth position of the ring can vary significantly without markedly affecting the herbicide’s activity [20]. Keto–enol tautomerism is a critical consideration in this class of compounds, affecting herbicidal activity [9]. As depicted in Figure 1, four tautomeric forms are possible: two ketoenolimine (KE–1 and KE–2), diketoenamine (DKE) and diketoimine (DKI). Instrumental analyses have indicated that the keto–enolic form (KE1) predominates over the diketo form, leading to the representation of general formulas in the literature as the keto–enolic form [3]. Another important aspect of these molecules is *E*–*Z* isomerization. Like other members of its family, profoxydim is marketed in the *E* isomer form. However, isomerization to the *Z* form can occur under various conditions. Additionally, profoxydim contains two chiral centers, and although a racemic mixture is typically employed in experiments and in the field, it is necessary to specify the diastereoisomer (RR=SS or RS=SR) when performing calculations. In this regard, Cervantes–Diaz et al. (2024) developed a chromatographic method for the separation of the four diastereoisomers of profoxydim, with the objective of quantifying profoxydim in rice samples [21].

Tautomerism and *E*/*Z* isomerism play pivotal roles in the reactivity of these compounds, making it crucial to identify the most stable molecular configurations across various environments. The primary aim of this study is to ascertain the most stable form under differing pH conditions through a dual approach combining both experimental investigations and theoretical analyses. Experimental work will pinpoint the most stable configurations, while theoretical calculations will provide detailed insights into the structure, shedding light on the factors that contribute to the stabilization of various isomers and tautomers. Gaining an in–depth understanding of the chemical structure will illuminate the diverse aspects of their fate and behavior across the different environmental compartments where the herbicide is encountered.

## 2. Material and Methods

### 2.1. Reagents and Solutions

The analytical standard of the herbicide profoxydim ((1EZ)–N–[(2RS)–2–(4–chlorophenoxy)propoxy]butanimidoyl)–3–hydroxy–5–[(3RS)–thian–3–yl]cyclohex–2–en–1–one) (98.8% purity), marketed as the E isomer and a mixture of the four stereoisomers RR/SS/RS/SR, was supplied by BASF. Its properties include a molecular weight of 466.0 g/mol, a log Pow of 3.7, a water solubility of 531 mg/L at 25 °C, a vapor pressure of 1.7 × 10^−1^ mPa at 20 °C, and a pKa of 5.91 at 20 °C [22].

Pesticide-grade and HPLC-grade solvents used for the LC mobile phase, such as acetonitrile, were purchased from Labscan (Stillorgan, Co., Dublin, Ireland) and formic acid (p.a.) was obtained from Merck (Darmstadt, Germany).

Ultrapure water, used for the LC mobile phase and aqueous solutions, was produced using a Millipore system (Milli–Q–50 18MΩ, Orsay, France).

Deuterated solvents (methanol MeOD, dimethyl sulfoxide [DMSO]–d6, benzene, and CDCl_3_) used for acquiring NMR spectra were sourced from Sigma Aldrich with a purity of 99%.

### 2.2. Isomerizacion Experiments

A 50 mg/L stock solutions of profoxydim was prepared by weighing 5 mg and dissolving in the minimum amount of methanol (MeOH approximately 1%), followed by the addition of ultrapure water to achieve the required volume. The standard solutions were stored in amber bottles at 4 °C in darkness. The stability of these solutions under these conditions was verified and demonstrated for at least 15 days. These solutions were used to prepare more diluted standard solutions. Herbicide solutions used for the isomerization experiments were prepared at a concentration of 10 mg/L by dissolving the appropriate volume of the stock solution in ultrapure water. This concentration was chosen to ensure detectable isomerization activity during the study.

Four 20 mL vials of the herbicide solution were prepared, to which formic acid was added to adjust the pH from neutral to acidic, specifically to a pH of 3. In addition, blank samples containing only the active substance were prepared to ensure a starting point of 100% product when conducting the assay. Two vials were kept at room temperature in darkness, while the other two vials were stored in a refrigerator at 4 °C. Aliquots of 50 µL from each of the four experiments were sampled once a day over a period of 30 days.

### 2.3. Chromatographic Analysis

The isomerization study of the herbicide profoxydim was conducted using an HPLC system (1100 series; Agilent Technologies, Palo Alto, CA, USA) coupled to a diode array detector (DAD). The analytical column was a C18 Waters Atlantis^®^ column (3 μm particle size, 4.6 mm × 150 mm) (Waters, Dublin, Ireland), equipped with an ODS guard column and maintained at 25 °C. Aliquots of 100 µL were injected into the HPLC system. The mobile phase consisted of a mixture of acetonitrile–water (90:10% *v*/*v*, with 0.1% formic acid), with a flow rate of 1 mL/min over 10 min. The detector was set to the maximum absorbance wavelength of profoxydim (230 nm). Under these conditions, the retention time was 3.5 min for *Z*–profoxydim and 6.5 min for *E*–profoxydim. The percentage ratios of *E*/*Z*–profoxydim were determined by measuring the peak areas.

### 2.4. NMR Experiments

NMR spectroscopy analyses of the herbicide were performed on a Bruker Avance AV–500 (500 MHz) spectrometer (Rheinstetten, Germany) for MeOD and DMSO, and in the case of CDCl_3_ and benzene, a Bruker AVIII–700 (700 MHz) was used. In the four solvents, tetramethylsilane (TMS) was used as an internal standard. Nuclear magnetic resonance (NMR) ^1^H and ^13^C, DEPT–135 (distortionless enhancement by polarization transfer 135 in 13C NMR) spectra were recorded and ^1^H chemical shifts were assigned using 1D and 2D 1H NMR COSY (correlation spectroscopy), while ^13^C resonances were assigned using 2D NMR HMBC (heteronuclear multiple-bond correlation) and HMQC (heteronuclear multiple-quantum correlation) techniques. The sample concentrations were of 10 mM in MeOD and DMSO. In CDCl_3_ and benzene, saturated solutions were used. Considering the Pesticide Manual [22], the solubility is supposed to be 5,31 mg/L. ^13^C NMR in CDCl_3_ and benzene could not be measured, given the low solubility in apolar solvents.

### 2.5. Computational Details

The conformational landscape of structures of each tautomer was obtained using a combination of semiempirical tight-binding methods [23] with a meta-dynamics-driven search algorithm, as implemented in the CREST software (Christchurch, UK), https://www.crestsoft.com/, accessed on 11 September 2024, [24]. An energy threshold of 6 kcal/mol was set. To obtain optimized molecular structures and more accurate free energies, the results were refined at density functional theory (DFT) level of theory by using the CENSO and ORCA programs (Maasgouw, The Netherlands) [25,26]. The optimization was carried out with the composite approach PBEh–3c [27] and def2–mSVP basis set, since it is a highly efficient method that performs well for the optimization of geometries. The final free energies were computed at ωB97x–D3 [28,29] with the def2–TZVPP basis set. In addition to the results in the gas phase, the water and octanol environments were simulated implicitly with the SMD model [30]. Solvation effects are considered in the calculation of the vibrational frequencies, and therefore in the entropic corrections and the free energies. The accuracy of the solvation models used in CENSO to predict free energies are thoroughly discussed [25]. We used Gibbs energies evaluated for each optimized conformer to select those ones that represent the 99% of the Boltzmann population for each solvent.

The theoretical NMR spectra were computed using the GIAO method [31] over the structures optimized with the CENSO program at B3LYP/6–311+G(d,p) level of theory [32,33] using the Gaussian 16 software suite of programs [34].

To obtain the preferred protonation site of each tautomer of profoxydim, we used the CREST tool for protonation site screening. We selected the most stable protomer for each tautomer, and we proceeded similarly to obtain the molecular conformations at DFT level, carrying out a calculation of the conformers using CREST followed by a refinement of free energies at DFT level with CENSO program.

### 2.6. QSAR Models

The acute and chronic toxicities (chV) of profoxydim’s tautomers were predicted for three aquatic organisms (fish, daphnia and green algae) using the US EPA, OECD and EU–approved QSAR ECOSAR software (version 2.2) [35].

The ECOSAR QSARs are based on a linear mathematical relationship between the predicted log Kow values and the corresponding log of the measured toxicity values (mmol/L) for a suite of training set chemicals within each class of interest. The studies collected for the training set chemicals in ECOSAR undergo an extensive data validation step to ensure appropriateness for inclusion in the model [36,37].

In the latest version of ECOSAR, the log Kow values for each training set chemical is predicted using the RS program from U.S. EPA’s Estimation Programs Interface Suite (EPI Suite™) model [38].

Acute toxicity parameters were demonstrated by the LC50 values (the concentration of the tested substance that is lethal to 50% of daphnia and fish after 48 h and 96 h of exposure, respectively), as well as by the EC50 values (the concentration of the tested substances for the growth deterrence of 50% of green algae after 96 h of exposure).

Chronic toxicity parameters are defined as the geometric mean of the non-observed-effect concentration (NOEC) and the lowest-observed-effect concentration (LOEC). This can be mathematically represented as: ChV = 10^([log (LOEC × NOEC)]/2).

The toxicity of the calculated values were classified based on the globally harmonized system of classification and the labeling of chemicals (GHS) (Table S1, supporting information) [39]. The estimation of toxicity provides beneficial information for the possible pre-emptive application of protocols to remove the desired pollutant from water and wastewater.

## 3. Results and Discussion

### 3.1. Structural Analysis

**Figure 2:**
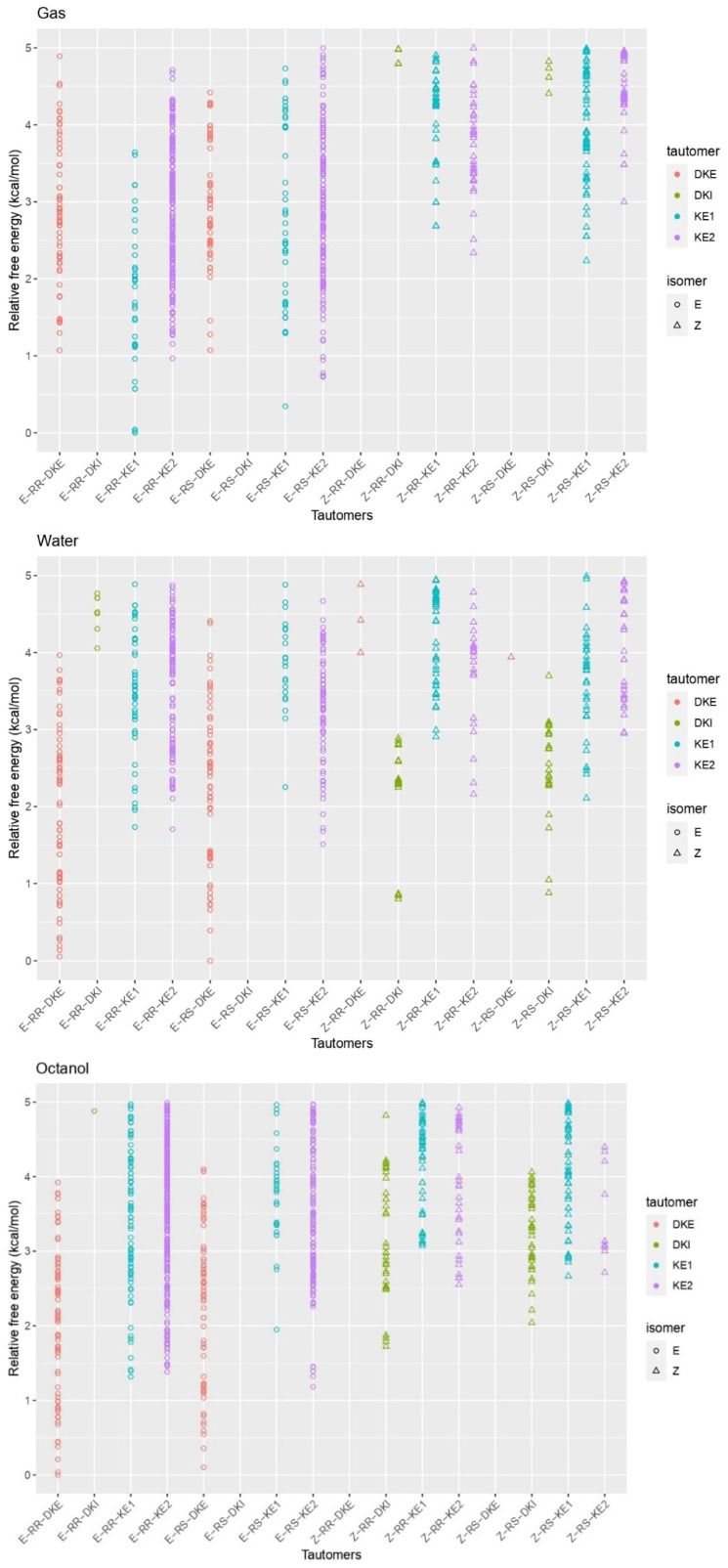
Relative stability of all the tautomer within the range of 5 kcal/mol.

In this study, both the chirality and *E*/*Z* isomerism of profoxydim were taken into account. Given that the RR/SS and RS/SR enantiomers exhibit identical properties in computational simulations, it was decided to investigate the eight tautomers of the RR and RS enantiomers. For each conformer, a variety of conformers within a 6 kcal/mol threshold set for CREST screening were examined. Due to profoxydim’s considerable rotational flexibility, more than two thousand conformers for each *E*/*Z* isomeric tautomer were analyzed and refined using the CENSO script across various computational steps. The study extended beyond gas-phase calculations to include simulations in two solvents: octanol and water. Figure 2 illustrates the relative energies among the most stable geometric conformations determined in the final step of the CENSO single point calculation at the wB97x–D3/def2–TZVPP level of theory simulated in water (In Figure S1, a similar figure is given in a larger energy range of 20 kcal/mol). The results in water as a solvent will be specifically discussed, as all experiments were conducted in water, and considering its effect will allow for a better understanding of its impacts on the tautomers/isomers’ stability. We will refer to the results from the gas phase and octanol whenever any differences are observed.

**Figure 3:**
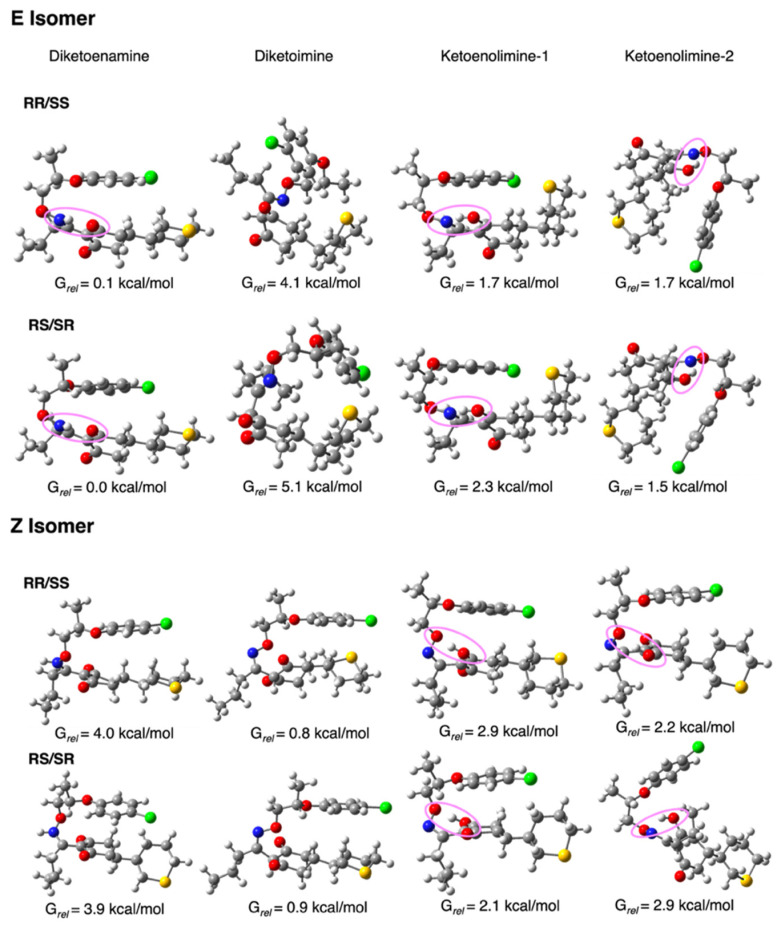
Optimized molecular geometries of the most stable conformers of each profoxydim’s tautomer. Relative free energy with respect to the most stable structure (*E*–diketoenamine–RS/SR) obtained in water as a solvent is given in kcal/mol. Hydrogen bonds are highlighted with a magenta oval. Color coding of atoms: hydrogen (white), carbon (gray), nitrogen (blue), oxygen (red), chlorine (green) and sulfur (yellow).

An overview of the three graphs in Figure 2 shows that the relative stability in terms of Gibbs free energy tends to suggest that the *E* isomer predominates in all media over the *Z* isomers. In the case of the gas phase (Figure 2, upper panel), the energy difference between the most stable of the former (*E*–RR–KE1) and the most stable of the latter (*Z*–RS–KE1) is approximately 2.2 kcal/mol. This gap narrows considerably when water is considered as a solvent. In this case, the E isomer remains the most stable, though in its ketonic form (*E*–RR–DKE), while the *Z* isomer is about 0.8 kcal/mol higher in its form (*Z*–RR–DKI). Furthermore, by analyzing the population of *E* isomers, we find a significant number of *E*–DKE conformers within a 1 kcal/mol energy range. For the *Z* isomer, only three conformers exist in the DKI form, which confirms the dominant presence of the *E* isomer. In the case of octanol, this difference enlarges to 1.8 kcal/mol (Figure 2, lower panel). Delving into the analysis of tautomers for each isomer, and focusing solely on the aqueous phase, it is observed that the two diastereoisomers RR/RS in the diketone (*E*–RR–DKE and *E*–RS–DKE) forms are the most stable, with a difference of approximately 1.7 kcal/mol from the closest enolic forms (*E*–RR–KE2 and *E*–RS–KE2; see Figure 3). Both forms are stabilized by a hydrogen bond. In the former, the NH group acts as the hydrogen bond donor to the carbonyl at position 3, whereas in the latter, the conversion of that same carbonyl to hydroxyl makes it a hydrogen bond donor to the nitrogen at position 7 (structures of Profoxydim are represented in 3D in Figure 3). In both tautomers, indeed, the hydrogen is movable through this bond from the oxygen to the nitrogen, since such barriers are typically low, generally favoring the amine.

**Figure 4:**
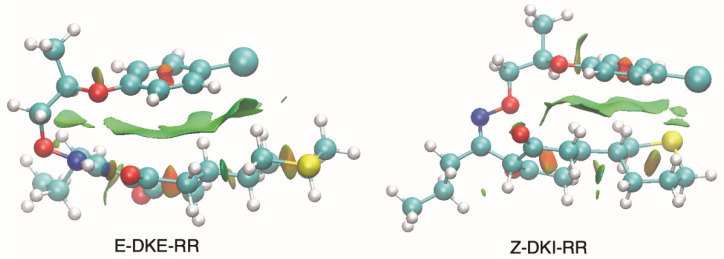
NCI analysis of the most stable conformer of each isomer of profoxydim in water.

In an in-depth comparison of all *Z/E* tautomers, the significance of stability due to non-covalent bonds becomes apparent. Observing Figure 3, we can see that the presence of phenoxy groups in profoxydim, across almost all conformers, is parallel, which is favored by π–π interactions. The most stable tautomer of the *E* isomer is stabilized, as mentioned earlier, by a hydrogen bond, but also by π–π interaction, as can be seen from the non-covalent interaction (NCI) analysis depicted in Figure 4. There is a clear dark blue coloration, indicating a strong HB interaction between NH and O, and a green color between the parallel fragments indicative of a weak interaction. Meanwhile, the most stable tautomer of the *Z* isomer, despite the absence of a hydrogen bond, has several interactions that favor its stability, reflected by the green markings between its various substituents (see Figure 4). This stability is accentuated when analyzing the results in the aqueous phase, considering the bulk water effect, which is not observed in the gas phase where the Z isomer is 2.2 kcal/mol higher (see Figure 2).

### 3.2. NMR Analysis

Polar solvents, such as water, act as external stabilizing agents for weaker hydrogen bonds, like those found in the *E*–diketoenamine (N–H–O) structure. This suggests that profoxydim, when marketed in aqueous solution as the *E* isomer, could actually be in the tautomeric *E*–DKE form. To determine which tautomer is present in different solvents, 1 D and 2D NMR spectra were performed in four solvents; two polar ones, MeOD (Figure S2) and DMSO (Figure S3), and two non-polar ones, CHCl_3_ (Figure S4) and benzene (Figure S5). In the latter two solvents, ^13^C could not be recorded due to the low solubility of profoxydim [22]. Theoretical chemical shifts were calculated for each of the previously proposed structures in order to compare them and find matches with the experimental values for the most stable tautomers. Table 1 and Table 2 presents the chemical shifts of profoxydim in MeOD and CDCl_3_, assigned to each atom based on the analysis of various techniques (refer to Figures S2 and S4 in the supporting information). Broadly, the comparison of the chemical shifts from the proton NMR spectra are similar between the theoretical and experimental results. The theoretical prediction error ranges between 0.05 and 1 ppm, depending on the position of the hydrogen analyzed. Experimentally, a hydrogen shift potentially involved in hydrogen bonding is observed around 14 ppm in CDCl_3_. This is consistent with the shifts observed in three hydrogen-bond stabilized tautomers (E–DKE, E–KE1, and E–KE2). In contrast, the E–DKI tautomer, which lacks hydrogen bonding, shows a theoretical hydrogen shift near 4 ppm, characteristic of a hydroxylic proton. This observation allows us to rule out the presence of the E–DKI tautomer in the solution. Therefore, two possibilities remain: profoxydim in its diketone form (E–DKE) and the ketoenol structures (E–KE1 and E–KE2), which can be distinguished in NMR by two significant features: the presence of an OH group in the enolic forms and its absence in the diketone form.

From a theoretical perspective, it has previously been suggested that enolic forms are more stable in the gas phase (see Figure 2), while diketone forms are more stable in aqueous environments. In the NMR spectra in CDCl_3_, a medium more akin to the gas phase, an acidic proton around 14 ppm, is observed and can be assigned to an OH group, thus confirming the presence of enolic forms, in good agreement with theoretical calculations (see Figure 2). In the NMR spectra obtained in polar solvents such as DMSO and MeOD, which are analogous to the aqueous phase conditions of the theoretical calculations, there is no presence of the proton at 14 ppm, confirming the predominance of the diketone form in these environments, in line with theoretical results. It is important to note that in these last two solvents, a DEPT–135 carbon–13 NMR analysis was conducted, confirming that the C2 atoms is quaternary (see Figure 1), definitively corroborating the presence of the E–DKE form, and discarding the other diketone form, E–DKI, in these environments. This conclusion aligns with the findings for other cyclohexanedione oxime herbicides, such as clethodim, in acetonitrile or chloroform [9].

### 3.3. Effect of pH

**Figure 5:**
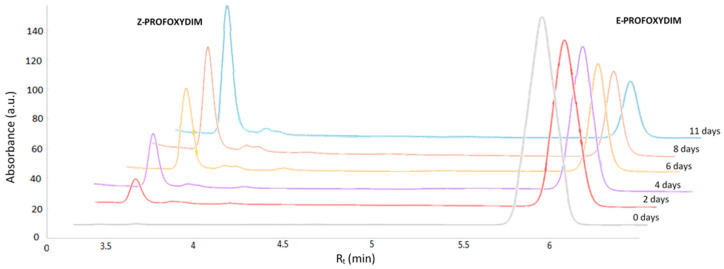
Superimposed chromatograms of the evolution of *E*/*Z* isomer of profoxydim at pH = 3.

Nowhere in the literature have we found a study that could reveal the significance of the *Z* isomer, since only the *E* isomer of profoxydim is marketed at 98% purity. This is indeed the analytical standard used in laboratories under normal conditions of pressure, temperature, and pH. However, in the natural environment where this herbicide is applied, conditions may vary, potentially altering the abundance of the isomers and, consequently, its efficacy. Our next question is: would the abundance of the *E*/*Z* isomers change with pH? How would such a change affect the different tautomers previously discussed? We have demonstrated, both experimentally and theoretically, that under normal conditions, the *E* isomer in its DKE form is the most predominant in the aqueous phase. To examine its behavior when the pH changes, an isomerization test of the herbicide in an acidic medium is conducted, as this type of herbicide is pH-dependent and tends to react more rapidly in acidic environments [40,41,42]. For this purpose, several chromatograms were recorded, tracking the evolution of the *E*/*Z* isomer over time. The results are presented in Figure 5. Table 3 lists the abundance of each isomer over time at pH = 3.

**Figure 6:**
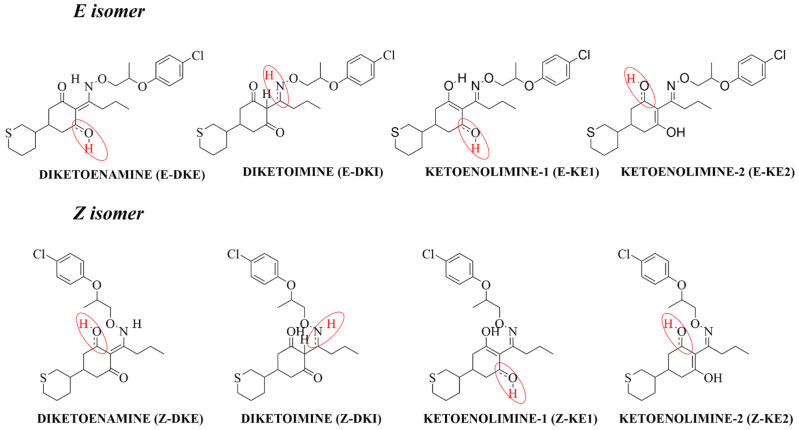
Positions considered for protonation in profoxydim molecules.

As depicted in Figure 5, isomerization from the *E* isomer to the *Z* isomer occurred over a period of 15 days, with a maximum of 70% of the *Z* product being formed. The assay was continued for 30 days to ensure that the *E*/*Z* ratio remained stable, which was confirmed. Now, the question arises: which *Z* conformer is the most abundant, and can these results be predicted theoretically? To tackle this question theoretically, the protomers of profoxydim were studied. The most stable position for protonation for each tautomer is depicted in Figure 6 (note that adding a proton to the molecule changes the tautomerism drastically. We should no longer talk of diketoenamine but ketoenolenamine in the case of DKE, and not of ketoenolimine but diolimine in the case of KE1; however, for consistency and to better compare these results with the previous results obtained from a neutral position, we have maintained the nomenclature and named protomers after the neutral structure that is protonated).

For each isomer/tautomer, there are five potential protonation sites: the two carbonyl oxygens at positions 1 and 3, the oxime’s oxygen or nitrogen, and lastly, the sulfur. All of these positions were initially considered. Protonation in S or protonation in both C=O groups in the case of DKI was 20 kcal/mol higher in energy than the most stable ones depicted in Figure 1, and was disregarded before conducting a CREST screening of all possible conformers. The relative stability for conformers were evaluated with the CENSO program for all selected protomers using the same protocol as the one described for neutral tautomers.

**Figure 7:**
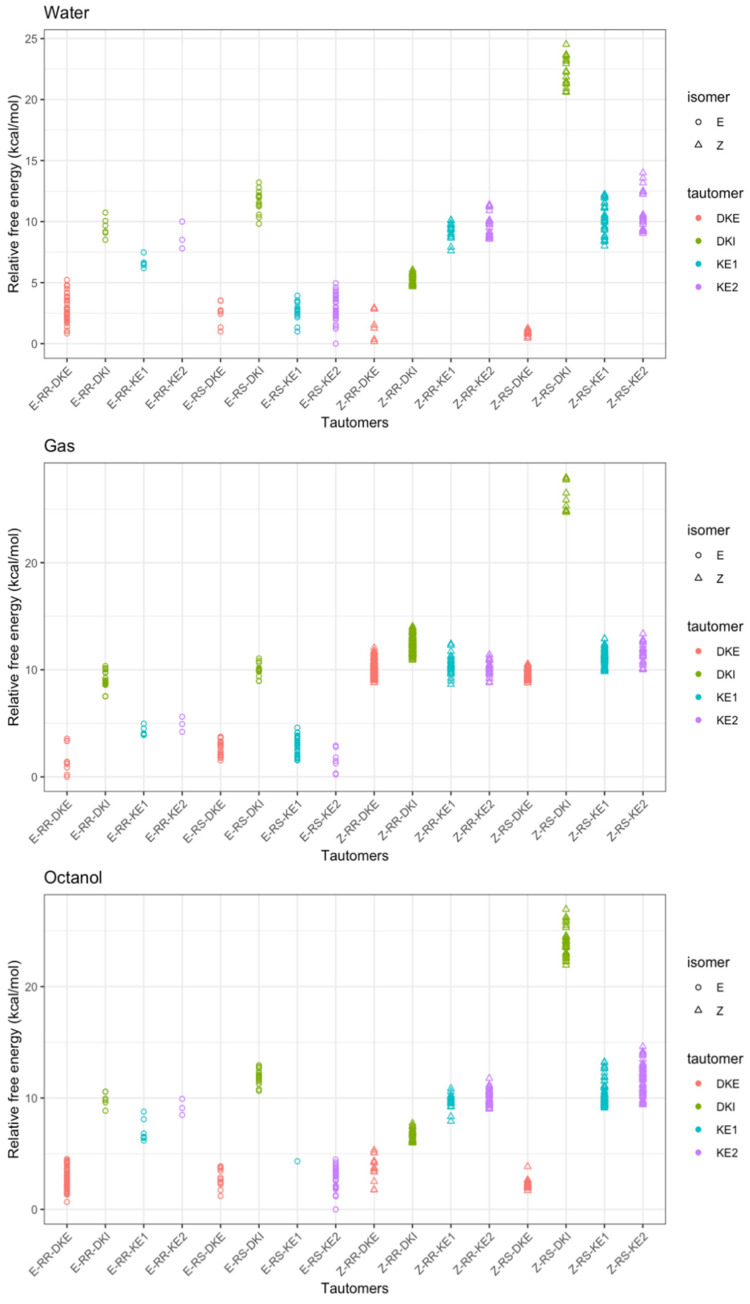
Relative stability of all the tautomers within the range of 25 kcal/mol (30 kcal/mol for gas and octanol).

Figure 7 presents the results for structures within a 25 kcal/mol stability range. A similar figure for a shorter energy range of 5 kcal/mol is given in Figure S6. It shows that the protomers from the E–RS–KE2 and Z–RR–DKE tautomers are competitive in stability. The most stable in both isomers are almost degenerate, with a difference of approximately 0.2 kcal/mol between the first and the second in aqueous phase (see Figure 7). The third most stable is the protomer from *Z*–RS–DKE, situated about 0.6 kcal/mol above the most stable. Significantly, in water, the population of low-lying conformers from the *Z* isomer becomes broader compared to that from the *E* isomer, aligning well with the experimental results shown earlier that at pH = 3; the presence of the *Z* isomer increases relative to the *E* isomer.

**Figure 8:**
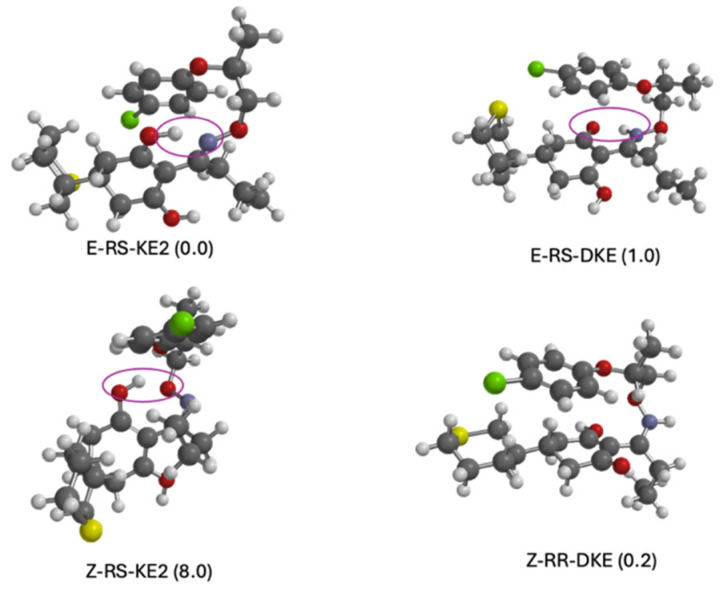
The optimized molecular geometries of the most stable protomers of the *E* compared with *Z* counterpart. Hydrogen bonds are highlighted with a magenta. Color coding of atoms: hydrogen (white), carbon (gray), nitrogen (blue), oxygen (red), chlorine (green) and sulfur (yellow).

Regarding the structure of the protomers, as depicted in Figure 8, we compared the most stable *E* (E–RS–KE2), the most stable Z (Z–RR–DKE) with their Z and E counterparts, respectively. The most stable *E* tautomer is stabilized by a hydrogen bond from OH acting as a hydrogen donor to N, while the other carbonyl at position 1 sustains protonation (Figure 8). In the case of the same tautomer for the *Z* isomer (Z–RS–KE2) the relative position of the N (due to the *Z* character) does not allow it to form this kind of hydrogen bond, and as the oxygen of the oxime is the one acting as acceptor, this hydrogen bond is less stable than the previous one. In the case of the most stable *Z* isomer (Z–RS–DKE), the large separation between the C=O at position 3 and the NH allows it to protonate in this carbonyl group, while for E–RS–DKE the two groups were too close to each other to allow for protonation in this site.

Analyzing the structure of profoxydim, there are five potential protonation sites: the two carbonyl oxygens at positions 1 and 3, the oxime’s oxygen or nitrogen, and lastly, the sulfonyl sulfur. However, our calculations show that with the exception of DKI tautomer, the carbonyl oxygens are the most probable centers. The relative stability analysis in terms of Gibbs free energy shows that the protomers from the E–RS–KE2 and Z–RR–DKE tautomers are competitive in stability.

Lastly, it is noteworthy that the population of the E isomer relative to the Z isomer changes depending on the solvent used for theoretical calculations. In the gas phase, the protomer originating from the E–DKE tautomer is the most stable, followed by the E–KE2 tautomer. In this scenario, all conformers from the Z isomer are more than 5 kcal/mol higher in energy than those from the *E* isomer (see Figure 7). In the case of octanol as solvent, only two protomers from the *Z* isomer appear, but they are 1.5 kcal/mol higher in energy than the most stable one.

### 3.4. QSAR Models

The significance of isomerization and tautomerization plays a crucial role in the effectiveness of profoxydim, as well as its toxicity in the environment where it may act. For this reason, studies have been conducted using the ECOSAR program to observe the impact that the molecular structures previously analyzed theoretically have on the environment and the living organisms within it. Through this study, we could verify whether different conformations of the same compound have varying effects on toxicity. The results are listed in Table 4.

The interpretation of the results presented in Table 4 indicates that the compound is very toxic, both in its isomeric and tautomeric forms, since the GHS classification (Table S1) indicates that any value below 1 is considered very toxic. If we go deeper into the results, Table 4 indicates that E/Z–DKE is the least toxic of the tautomers studied. It requires 0.376 mg/L to eliminate 50% of the fish population, 0.068 mg/L for 50% of the Daphnia population, and 0.025 mg/L for 50% of the algae population. For DKI, KE1, and KE2 tautomers, only half the amount is lethal for all trophic forms of life in aquatic environments. Based on the aforementioned results, which demonstrate that profoxydim predominantly exists in the E–diketoenamine form, it suggests that its toxicity would be the lowest of all possible forms. However, when the environment is acidic, the most stable protomers are E–KE2 and Z–DKE (Figure 7 and Figure 8), and thus the toxicity could increase for all the species studied, since the KE2 tautomer presents more toxicity than DK1 and DKE.

## 4. Conclusions

In this study, we have conducted a detailed investigation of the chemical structure of profoxydim from both theoretical and experimental perspectives for the first time. Although the E–isomer of profoxydim is primarily commercialized, it was unclear which tautomer was predominant under different environmental conditions. Through a rigorous exploration of various tautomers and their potential conformers at a high level of theory, we have successfully identified all possible structures. This analysis also extended to the tautomers/conformers of the Z–isomer. Our results indicate that the enolic form is more prevalent in the gas phase, while the ketonic form dominates in aqueous environments. This observation was corroborated by proton and carbon–13 NMR spectroscopy analysis of the E–isomer in solvents like MeOD, DMSO, CDCl_3_ and benzene, which simulate the conditions of the theoretical calculations.

Furthermore, we observed the conversion of the E–isomer to the Z–isomer under acidic conditions, a phenomenon that could impact the reactivity of commercialized profoxydim in natural environments. To investigate this, theoretical calculations were performed on the protonated forms of all previously studied tautomers/conformers, confirming this conversion and its potential ease of occurrence. Experimental data confirm that the E and Z forms become degenerate in acidic environments, while in neutral conditions, the E form is the most dominant. Additionally, QSAR models were applied to assess the toxicity of the different tautomers, revealing significant variability depending on the specific tautomer. This aspect is crucial for understanding how different tautomeric forms can influence the bioactivity and toxicity profiles of the profoxydim.

Despite the fact that the optimal structure for maximum activity has not yet been discovered, especially considering that these herbicides were developed without any rational structure-based optimization [1], it is likely that the findings from this structural study will significantly contribute to the development of cyclohexanedione chemistry. These advancements could enable the identification of more active molecules, including compounds capable of controlling resistant grass weeds, and provide a basis for the rational design of new herbicides that better address current agricultural challenges.

## Figures and Tables

**Table 1:** Experimental and calculated ^1^H–NMR chemical shifts for *E*–profoxydim in CH_3_OD and CDCl_3_.

	Exp. δ	Exp. δ	E–DKE	E–DKI	E–KE1	E–KE2
	(ppm) CH_3_OD	(ppm) CDCl_3_				
Diketone ring						
H4a	2.19	2.19	1.78	1.94	2.13	2.54
H4b	2.10	2.13	2.23	3.09	2.67	2.37
H5	1.99	2.03	0.50	3.45	3.18	1.38
H6a	2.36	2.41	2.23	2.80	2.52	2.60
H6b	2.32	2.38	1.63	2.01	1.88	2.64
C–chain						
H8a	2.36	2.4	2.90	2.06	2.13	2.77
H8b	2.42	2.43	2.47	2.65	1.65	2.72
H9a,b	1.37	1.50	1.53	1.87	1.31	1.78
			1.64	0.90	1.40	1.13
H10a	0.85	0.95	1.11	–0.07	0.81	0.15
H10b	0.82	0.93	0.97	–0.96	0.75	–0.72
H10c	0.80	0.92	0.92	–0.02	1.00	0.23
Aromatic						
H12	6.99	6.89	6.94	7.09	7.23	7.07
H13	7.21	7.24	7.43	7.45	7.26	7.37
H15	7.21	7.24	7.31	7.54	7.81	7.42
H16	6.99	6.89	6.95	7.32	7.15	7.10
C–chain (O–O)						
H17	4.93–4.71	4.68–4.57	4.87	5.25	5.15	5.19
H18	4.20–4.04	4.26–4.16	4.30	4.24	4.16	4.21
			4.15	4.29	3.97	4.24
H19a,b,c	1.30	1.36	0.80	1.52	1.10	0.45
			0.96	0.82	1.48	0.95
			1.60	0.99	1.10	1.03
Thiane ring						
H20	1.59	1.61	0.95	1.34	1.39	1.51
H21a	2.06	2.08	1.09	1.84	1.78	0.80
H21b	1.67	1.60	1.82	1.40	1.37	1.45
H22a	2.50	2.49	1.92	1.79	2.03	1.98
H22b	2.40	2.46	1.45	1.55	1.40	0.97
H23a	1.88	1.86	2.72	2.86	2.53	2.55
H23b	1.20	1.19	2.33	2.90	2.91	2.39
H24a	2.40	2.42	2.61	2.71	2.76	2.11
H24b	2.60	2.61	2.53	3.00	2.95	2.52
Moving H						
NH OH H2	–	14.2	13.43 (N–H)	3.74 (H2)	14.57 (O–H)	13.62 (O–H)

**Table 2:** Experimental and calculated ^13^C–NMR chemical shifts for *E*–profoxydim in CH_3_OD.

	Exp. δ	E–DKE	E–DKI	E–KE1	E–KE2
	(ppm) CH_3_OD				
Diketone ring					
C1	193.38	204.2	220.7	205.2	187.1
C2	110.7	110.4	78.1	112.0	116.4
C3	193.3	206.4	224.7	192.9	204.9
C4	39.54	44.0	45.8	37.1	43.5
C5	38.82	38.6	28.1	31.9	38.1
C6	39.61	44.4	45.8	44.0	34.4
C–chain					
C7	161.70	176.4	167.4	168.6	168.5
C8	32.16	33.6	30.3	33.5	28.3
C9	18.86	23.7	19.6	22.8	20.9
C10	13.73	13.5	11.0	13.3	14.8
Aromatic					
C11	157.0	160.2	161.6	163.5	161.2
C12	117.6	115.6	122.6	125.6	114.3
C13	129.2	134.2	133.7	134.1	134.1
C14	125.3	133.4	132.3	135.3	132.8
C15	129.2	133.5	133.7	133.4	134.7
C16	117.6	122.6	116.7	123.3	123.2
C–chain (O–O)					
C17	73.0	70.2	75.4	75.7	71.3
C18	76.2	76.9	79.4	78.9	77.4
C19	16.36	12.3	13.1	15.6	13.0
Thiane ring					
C20	42.60	45.2	42.8	39.6	39.2
C21	28.01	30.4	28.9	28.4	31.1
C22	28.50	30.7	24.0	22.5	32.7
C23	29.87	33.2	34.9	34.1	33.5
C24	31.48	35.5	35.6	35.6	37.5

**Table 3:** Experimental values of the evolution of *E*/*Z* isomer of profoxydim at pH = 3.

Time (Days)	0	1	2	3	4	5	6	7	8	11	13	15	30
% Z–Profoxydim	1	8	13	24	25	30	35	42	45	56	62	70	70
% E–Profoxydim	99	92	87	76	75	70	65	58	55	44	38	30	30

**Table 4:** Prediction of acute and chronic toxicity for the different tautomers using the ECOSAR program.

Tautomers	Acute Toxicity [mg/L]			Chronic Toxicity (ChV) [mg/L]		
	Fish (LC_50_)	Daphnid (LC_50_)	Algae (EC_50_)	Fish	Daphnid	Algae
E/Z–DKE	0.376	0.068	0.025	0.0061	0.0084	0.0110
E/Z–DKI	0.163	0.032	0.010	0.0022	0.0041	0.0047
E/Z–KE1	0.160	0.031	0.0098	0.0021	0.0041	0.0046
E/Z–KE2	0.160	0.031	0.0098	0.0021	0.0041	0.0046

## Data Availability

The original contributions presented in the study are included in the article/Supplementary Material, further inquiries can be directed to the corresponding author/s.

## Supplementary Materials

The following supporting information can be downloaded at: https://www.mdpi.com/article/10.3390/molecules29184371/s1, Figure S1: Relative stability of all the tautomer within the range 10–20 kcal/mol. Figure S2: NMR spectra of profoxydim in MeOD. (a)^1^H-NMR. (b) ^13^C-NMR. (c) ^13^C-NMR dept. (d) HMQC. (e) HMBC. (f) COSY. Figure S3: NMR spectra of profoxydim in DMSO. (a) 1H-NMR. (b) 13C-NMR. (c) 13C-NMR dept. (d) HMQC. (e) COSY. Figure S4: NMR spectra of profoxydim in CDCl_3_. (a) ^1^H-NMR. (b) ^13^C-NMR. Figure S5: NMR spectra of profoxydim in benzene. ^1^H-NMR. Figure S6: Stability of all the protomers considered for the different isomer/tautomer within a range of 5 kcal/mol. Table S1: Classification scheme for substances hazardous to the aquatic environment according from the Globally Harmonized System of Classification and Labelling of Chemicals (GHS).
