# Supplementary material for: Profoxydim in Focus: A Structural Examination of Herbicide Behavior in Gas and Aqueous Phases
Source: Molecules. 2024 Sep 14;29(18):4371. doi: 10.3390/molecules29184371 (PMC11433996; doi:10.3390/molecules29184371)
Supplement: Supplementary file 1 [file molecules-29-04371-s001.zip › molecules-3190602-supplementary.pdf]

Profoxydim in Focus: A Structural Examination of Herbicide Behavior in Gas and Aqueous Phases

María Cobos-Escudero [1,2], Paula Pla [2], Álvaro Cervantes-Diaz [1], José Luis Alonso-Prados [1], Pilar Sandín-España [1,*], Manuel Alcamí [2,3,4] and Al Mokhtar Lamsabhi [2,3,*]

[1] Unit of Plant Protection Products, Instituto Nacional de Investigación y Tecnología Agraria y Alimentaria (INIA-CSIC), Carretera de La Coruña Km. 7, 28040 Madrid, Spain; maria.cobos@inia.csic.es (M.C.-E.); alvaro.cervantes@inia.csic.es (Á.C.-D.); prados@inia.csic.es (J.L.A.-P.)

[2] Departamento de Química, Facultad de Ciencias, M13, Universidad Autónoma de Madrid, 28049 Madrid, Spain; paula.pla@uam.es (P.P.); manuel.alcami@uam.es (M.A.)

[3] Institute for Advanced Research in Chemical Sciences (IAdChem), Universidad Autónoma de Madrid, 28049 Madrid, Spain

[4] Instituto Madrileño de Estudios Avanzados en Nanociencias (IMDEA–Nanociencia), 28049 Madrid, Spain

* Correspondence: sandin@inia.csic.es (P.S.-E.), mokhtar.lamsabhi@uam.es (A.M.L.)

Supporting Information:

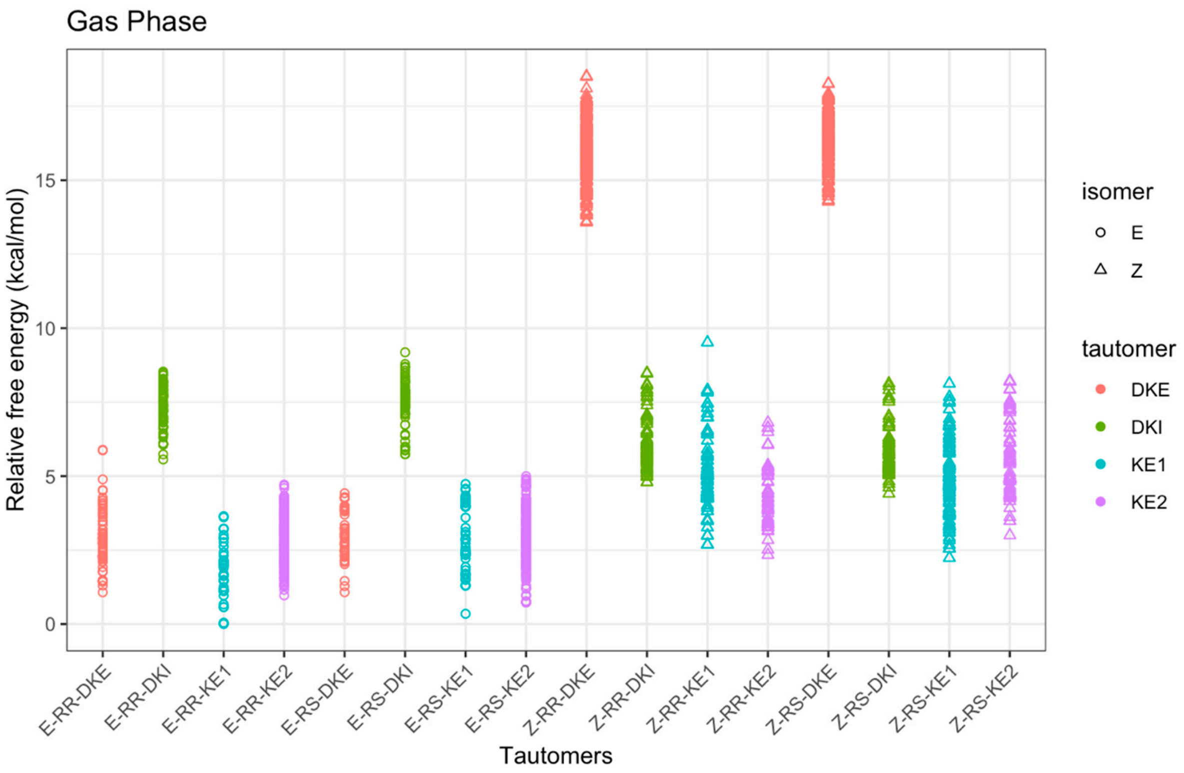

Gas Phase
Relative free energy (kcal/mol)
0
5
10
15
E-RR-DKE
E-RR-DKI
E-RR-KE1
E-RR-KE2
E-RS-DKE
E-RS-DKI
E-RS-KE1
E-RS-KE2
Z-RR-DKE
Z-RR-DKI
Z-RR-KE1
Z-RR-KE2
Z-RS-DKE
Z-RS-DKI
Z-RS-KE1
Z-RS-KE2
Tautomers
isomer
E
Z
tautomer
DKE
DKI
KE1
KE2

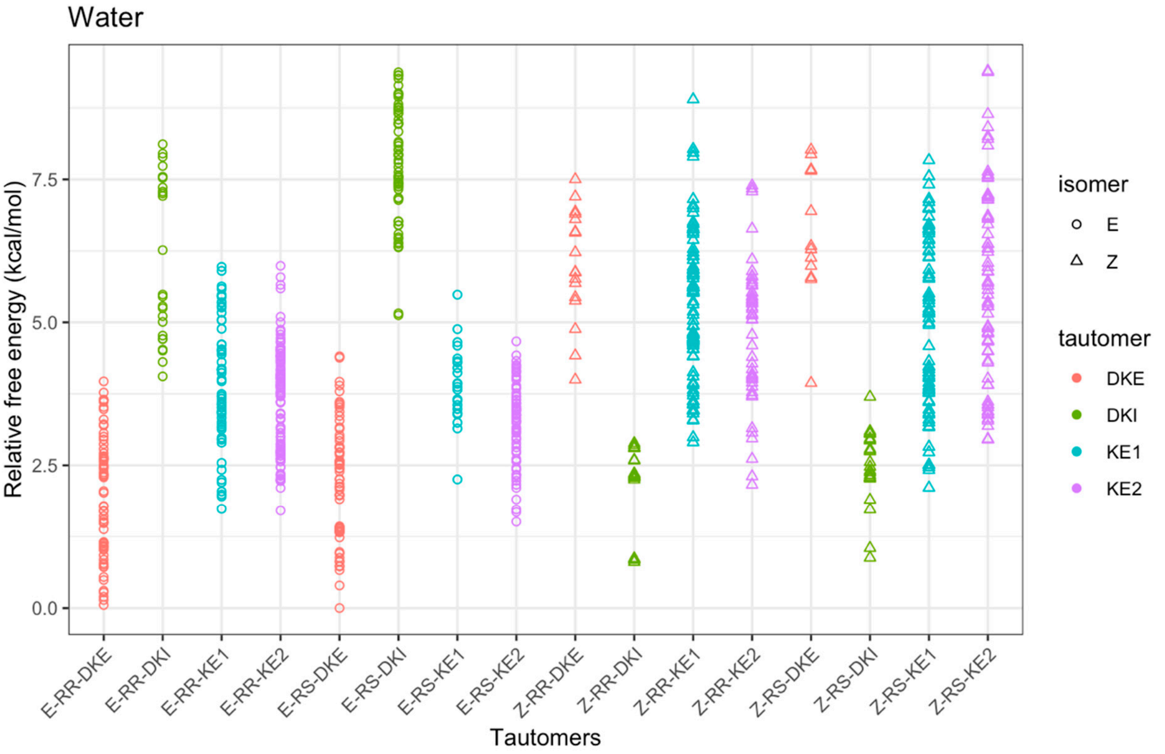

Water
Relative free energy (kcal/mol)
0.0
2.5
5.0
7.5
E-RR-DKE
E-RR-DKI
E-RR-KE1
E-RR-KE2
E-RS-DKE
E-RS-DKI
E-RS-KE1
E-RS-KE2
Z-RR-DKE
Z-RR-DKI
Z-RR-KE1
Z-RR-KE2
Z-RS-DKE
Z-RS-DKI
Z-RS-KE1
Z-RS-KE2
Tautomers
isomer
E
Z
tautomer
DKE
DKI
KE1
KE2

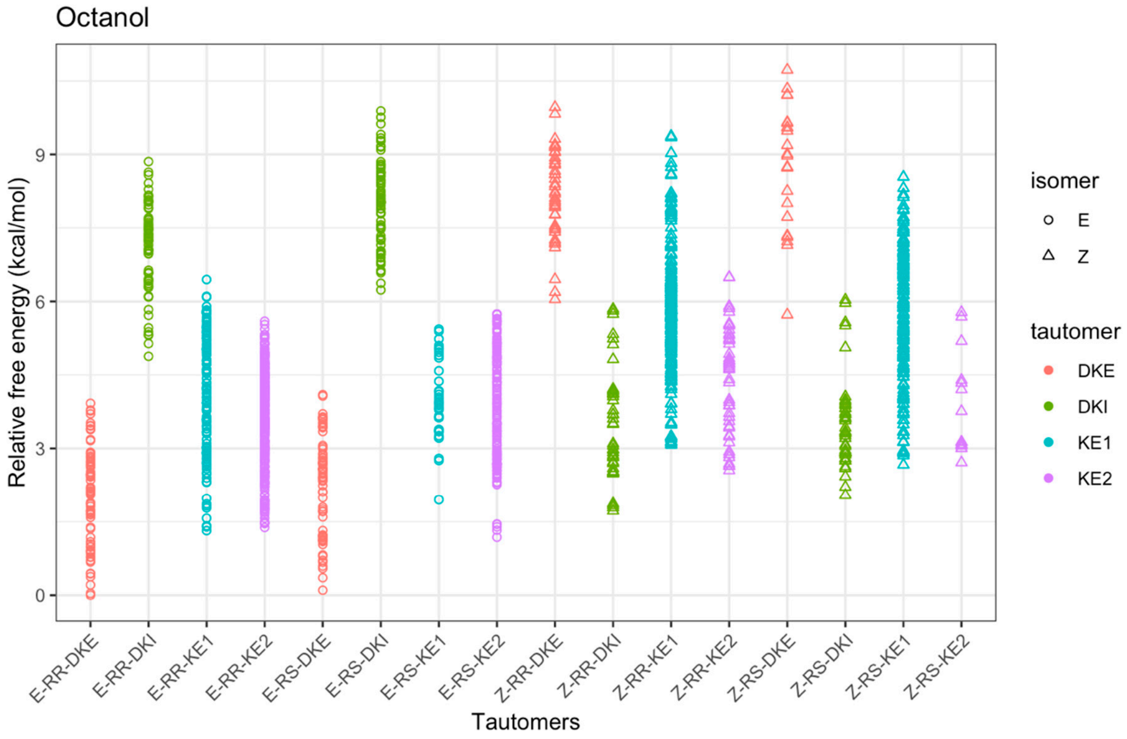

Figure S1. Relative stability of all the tautomer within the range 10-20 kcal/mol

a)

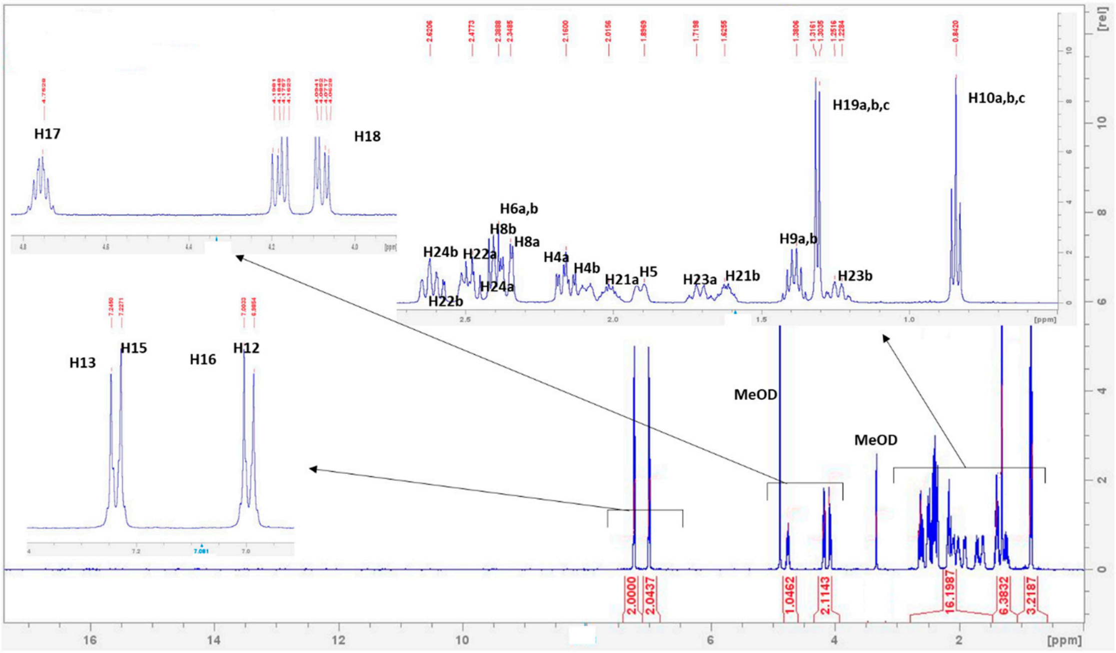
H17
H18
H19a,b,c
H10a,b,c
H6a,b
H8b
H8a
H24b
H22a
H4a
H4b
H24a
H22b
H21a
H5
H23a
H21b
H9a,b
H23b
H13
H15
H16
H12
MeOD
MeOD
2.0000
2.0437
1.0462
2.1143
16.1987
6.3832
3.2187
[ppm]
[rel]

b)

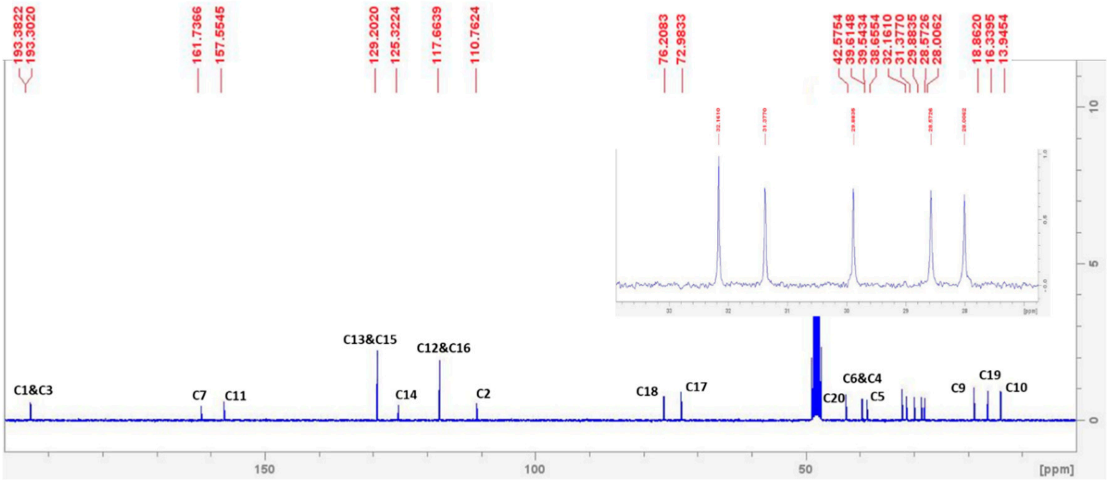
193.3822
193.3020
161.7366
157.5545
129.2020
125.3224
117.6639
110.7624
76.2083
72.9833
42.5754
39.6148
39.5434
38.6554
32.1610
31.3770
29.8835
28.5726
28.0062
18.8620
16.3395
13.9454
C1&C3
C7
C11
C13&C15
C14
C12&C16
C2
C18
C17
C20
C6&C4
C5
C9
C19
C10
[ppm]

c)

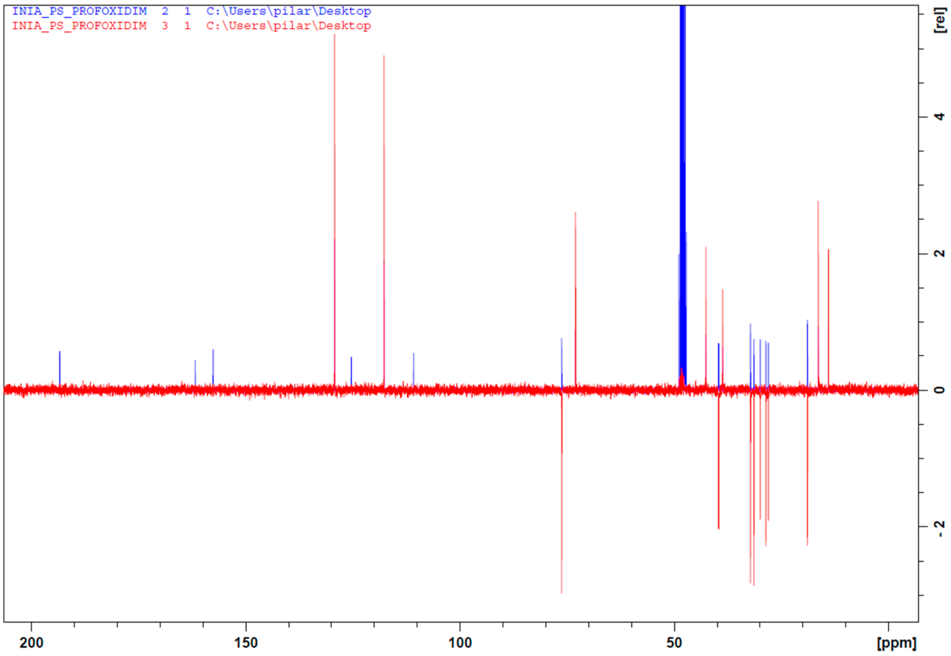
INIA_PS_PROFOXIDIM 2 1 C:\Users\pilar\Desktop
INIA_PS_PROFOXIDIM 3 1 C:\Users\pilar\Desktop
[rel]
4
2
0
- 2
200
150
100
50
[ppm]

d)

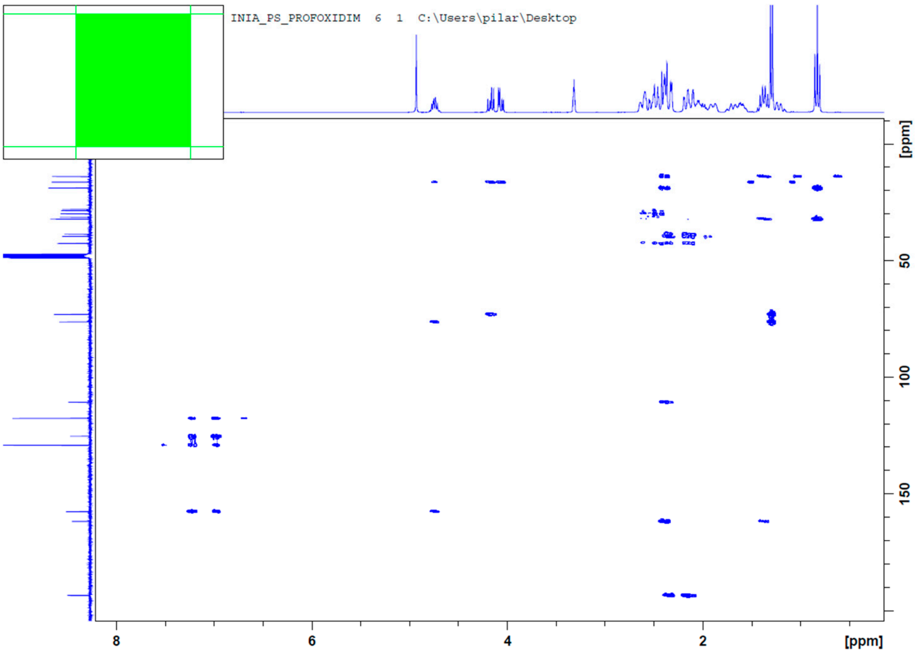
INIA_PS_PROFOXIDIM 6 1 C:\Users\pilar\Desktop
[ppm]
50
100
150
8
6
4
2
[ppm]

e)

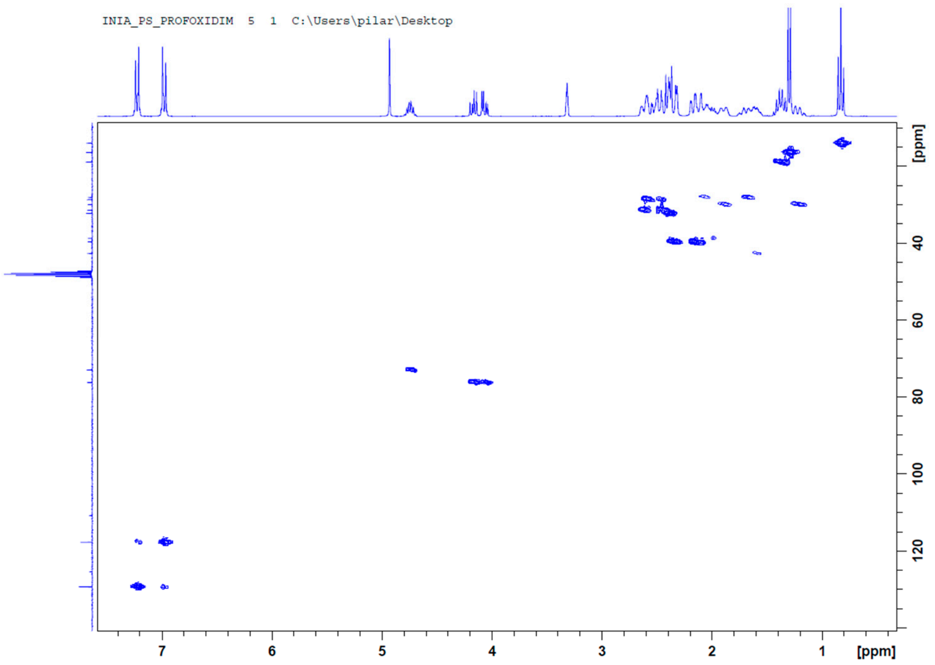

f)

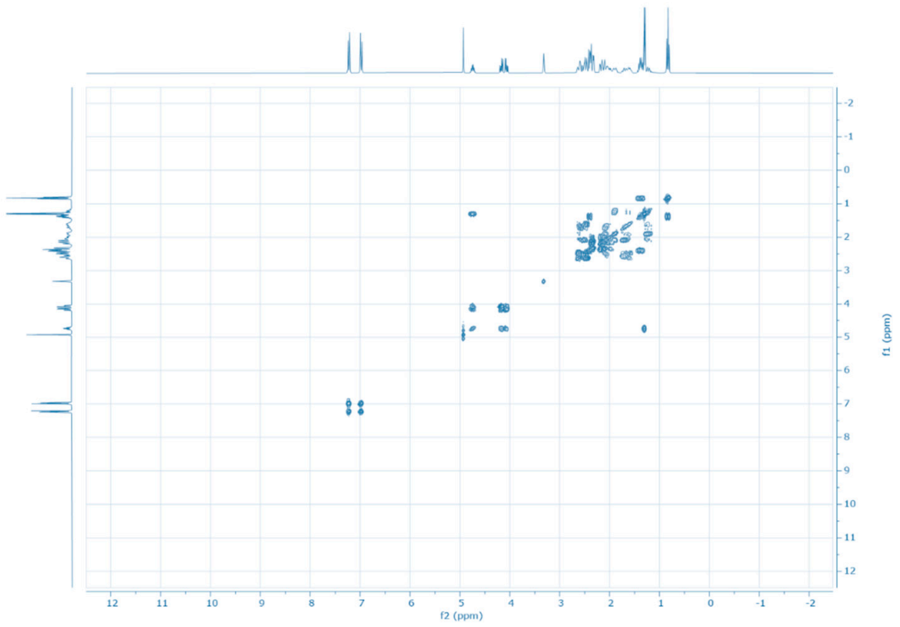

Figure S2. NMR spectra of profoxydim in MeOD. a)^{1}H-NMR. b) ^{13}C-NMR. c) ^{13}C-NMR dept. d) HMQC. e) HMBC. f) COSY.

a)

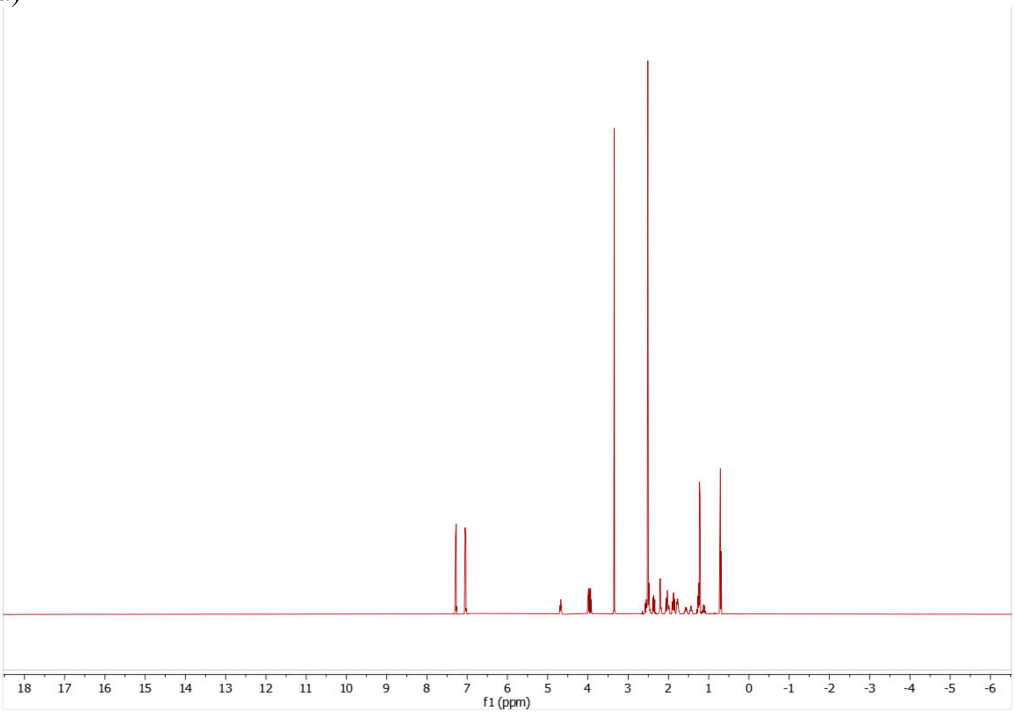

18 17 16 15 14 13 12 11 10 9 8 7 6 5 4 3 2 1 0 -1 -2 -3 -4 -5 -6
f1 (ppm)

b)

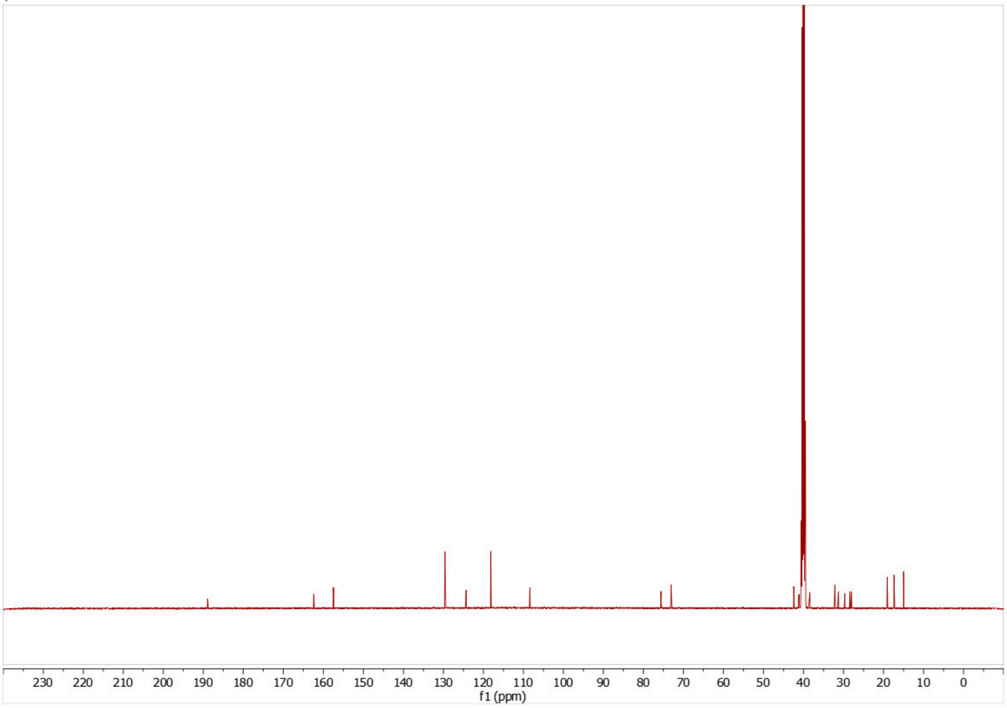

230 220 210 200 190 180 170 160 150 140 130 120 110 100 90 80 70 60 50 40 30 20 10 0
f1 (ppm)

c)

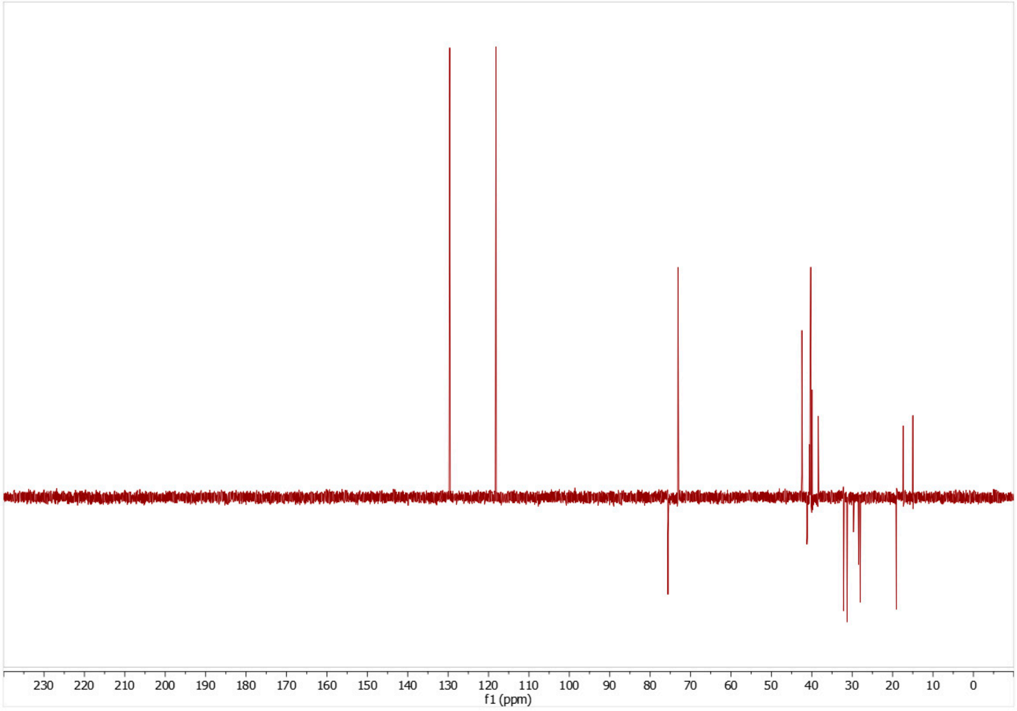
230 220 210 200 190 180 170 160 150 140 130 120 110 100 90 80 70 60 50 40 30 20 10 0
f1 (ppm)

d)

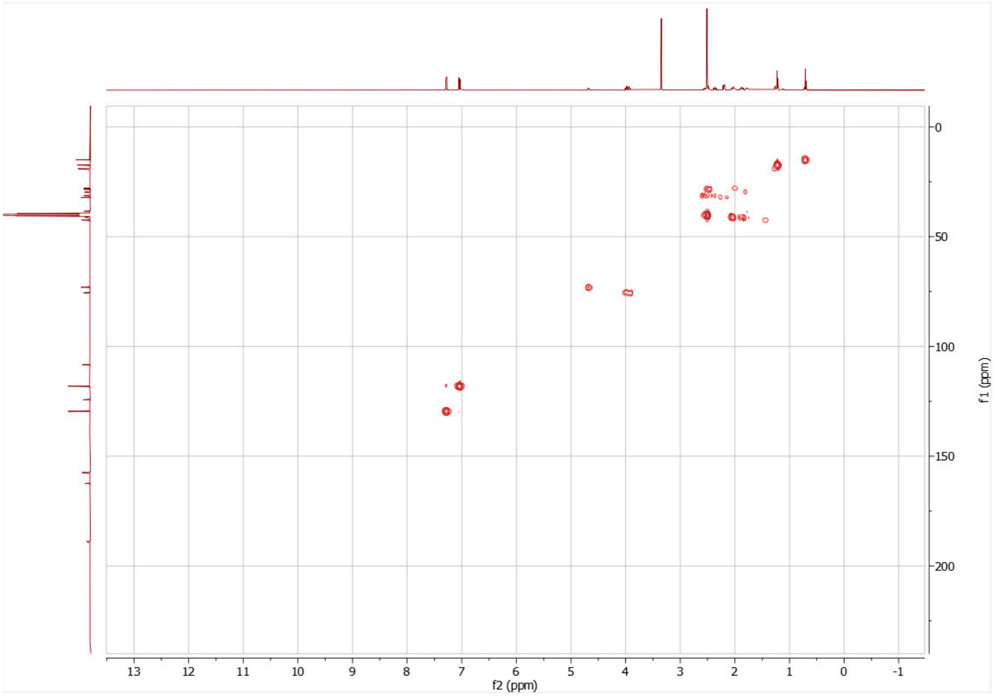
13 12 11 10 9 8 7 6 5 4 3 2 1 0 -1
f2 (ppm)
0 50 100 150 200
f1 (ppm)

e)

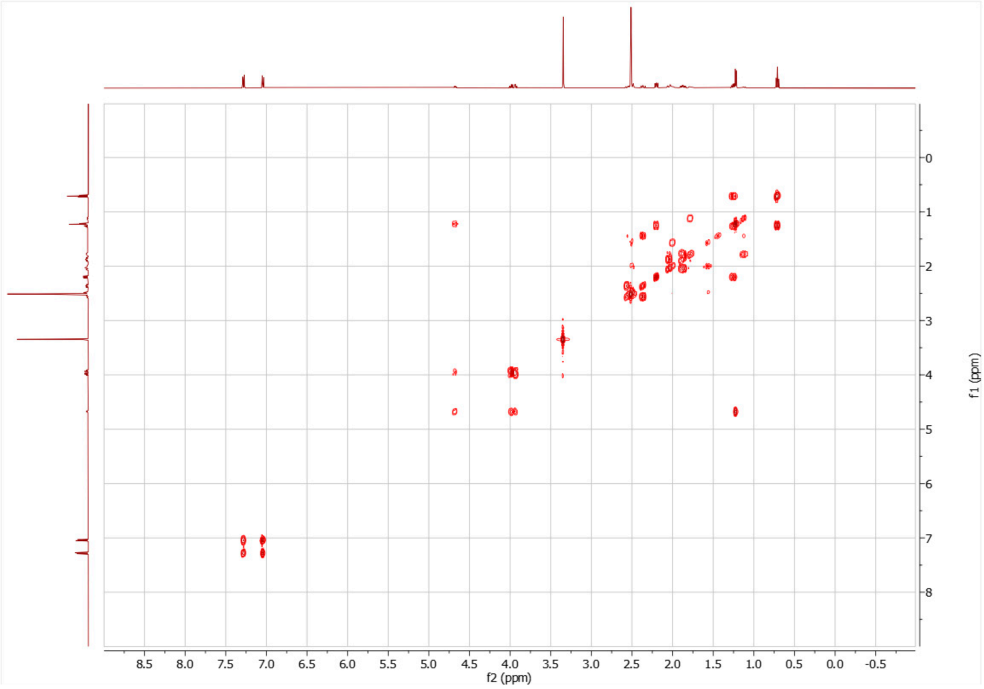

Figure S3. NMR spectra of profoxydim in DMSO. a) ^{1}H-NMR. b) ^{13}C-NMR. c) ^{13}C-NMR dept. d) HMQC. e) COSY.

a)

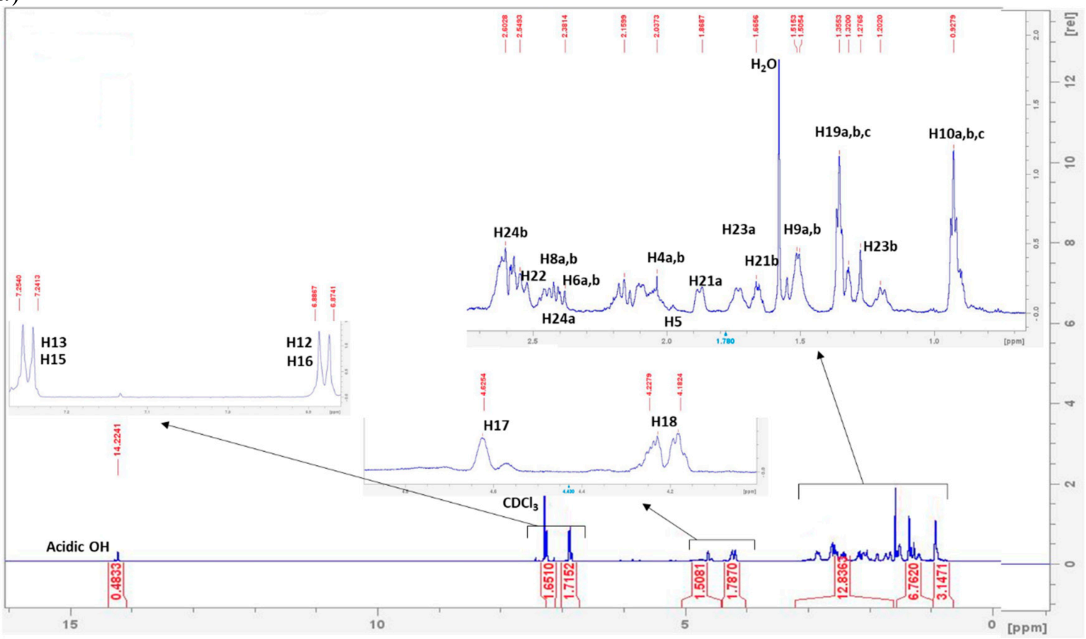

b)

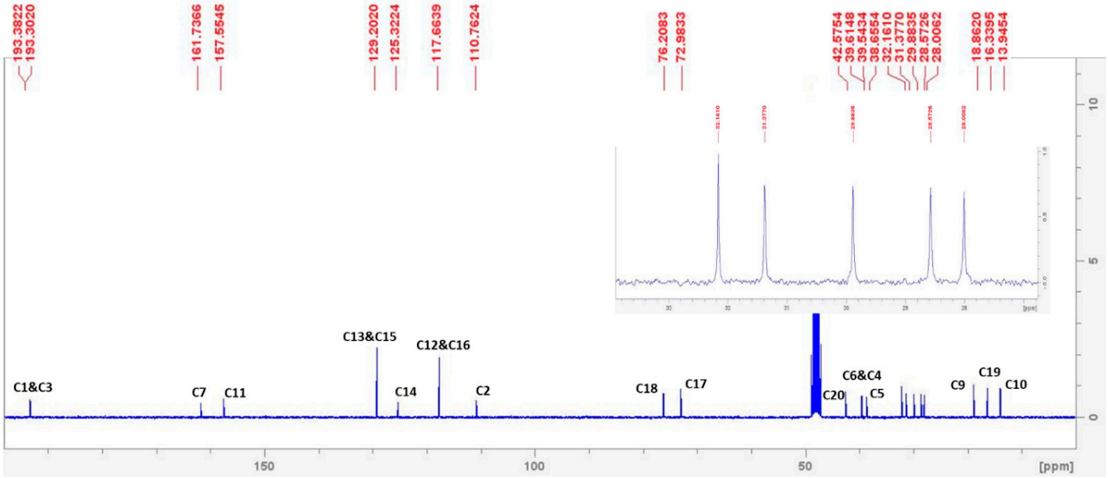

Figure S4. NMR spectra of profoxydim in $CDCl_3$. a) ^{1}H-NMR.b) ^{13}C-NMR.

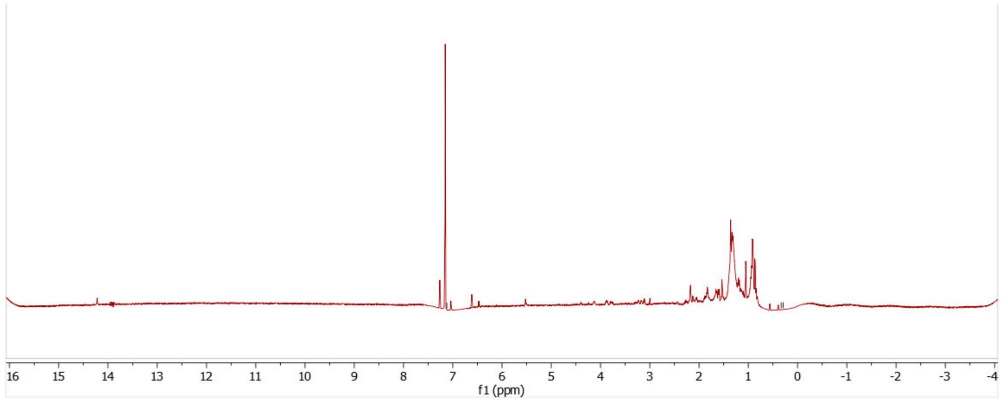

Figure S5. NMR spectra of profoxydim in benzene. ^{1}H-NMR.

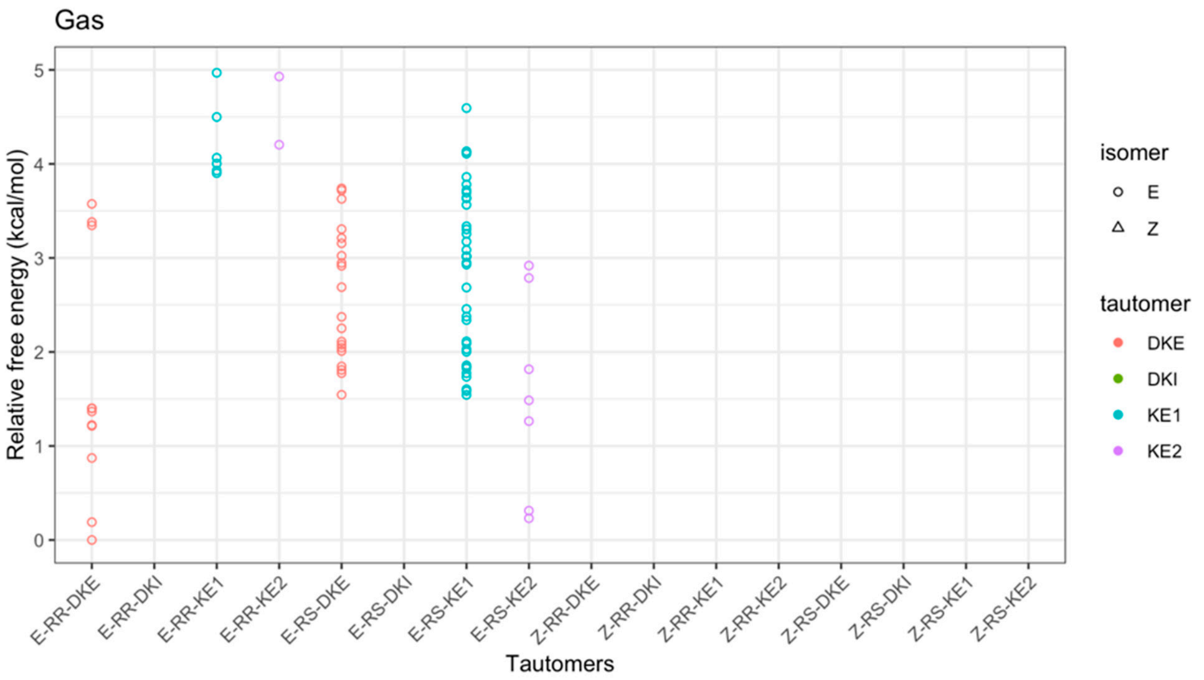
Gas
Relative free energy (kcal/mol)
0
1
2
3
4
5
E-RR-DKE
E-RR-DKI
E-RR-KE1
E-RR-KE2
E-RS-DKE
E-RS-DKI
E-RS-KE1
E-RS-KE2
Z-RR-DKE
Z-RR-DKI
Z-RR-KE1
Z-RR-KE2
Z-RS-DKE
Z-RS-DKI
Z-RS-KE1
Z-RS-KE2
Tautomers
isomer
E
Z
tautomer
DKE
DKI
KE1
KE2

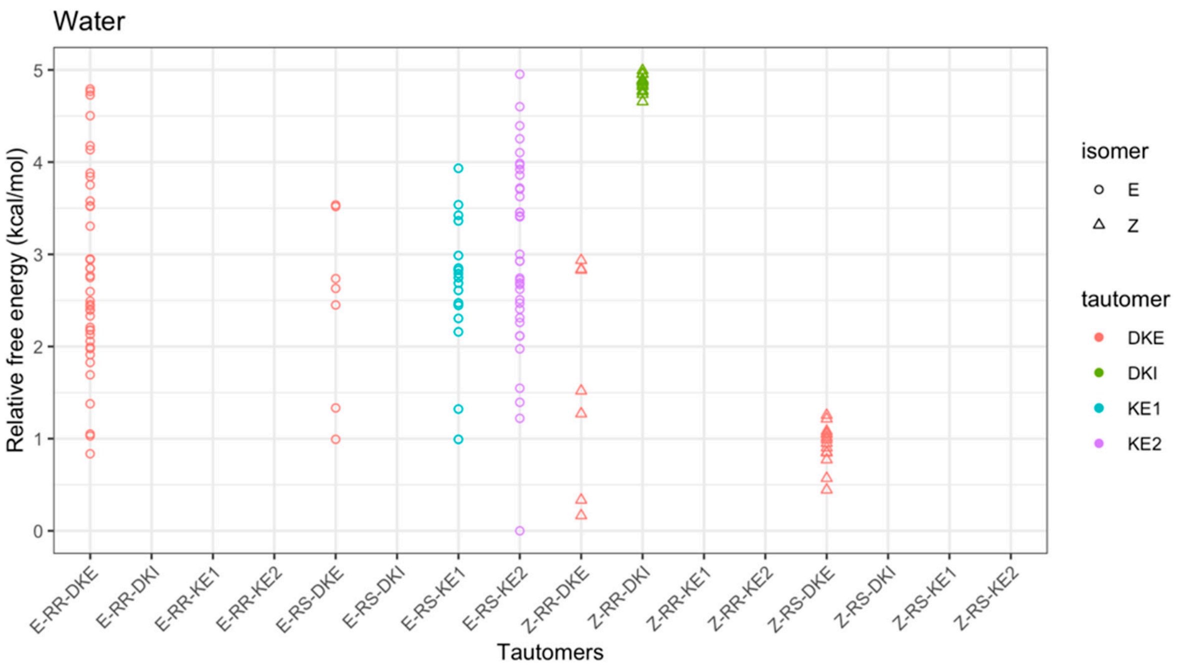
Water
Relative free energy (kcal/mol)
0
1
2
3
4
5
E-RR-DKE
E-RR-DKI
E-RR-KE1
E-RR-KE2
E-RS-DKE
E-RS-DKI
E-RS-KE1
E-RS-KE2
Z-RR-DKE
Z-RR-DKI
Z-RR-KE1
Z-RR-KE2
Z-RS-DKE
Z-RS-DKI
Z-RS-KE1
Z-RS-KE2
Tautomers
isomer
E
Z
tautomer
DKE
DKI
KE1
KE2

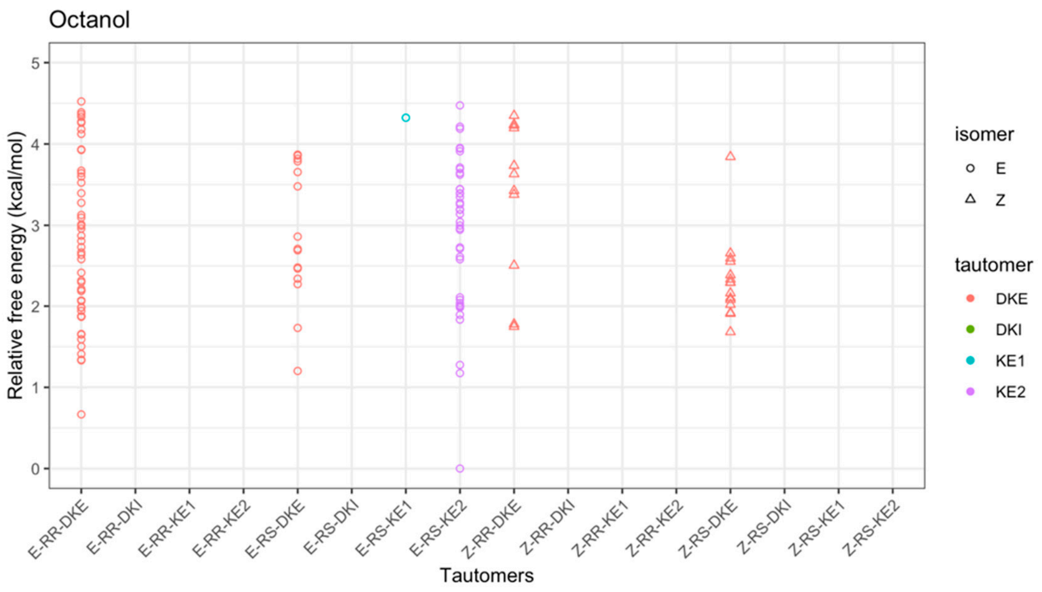

Figure S6: Stability of all the protomers considered for the different Isomer/tautomer within a range of 5 kcal/mol

Table S1: Classification scheme for substances hazardous to the aquatic environment according from the Globally Harmonized System of Classification and Labelling of Chemicals (GHS).

Toxicity	Category 1<br>**Very toxic**	Category 2<br>**Toxic**	Category 3<br>**Harmful**	Category 4<br>**Not Harmful**
Acute toxicity	$LC_{50}/EC_{50} \leq 1 mg/L$	$1 mg/L < LC_{50}/EC_{50} \leq 10 mg/L$	$10 mg/L < LC_{50}/EC_{50} \leq 100 mg/L$	$LC_{50}/EC_{50} > 100 mg/L$
Chronic toxicity	$ChV \leq 0.01 mg/L$	$0.01 mg/L < ChV \leq 0.1 mg/L$	$0.1 mg/L < ChV \leq 1 mg/L$	$ChV > 1 mg/L$
